# Supplementary material for: Pneumocystis spp. in Pigs: A Longitudinal Quantitative Study and Co-Infection Assessment in Austrian Farms
Source: J Fungi (Basel). 2021 Dec 31;8(1):43. doi: 10.3390/jof8010043 (PMC8779942; doi:10.3390/jof8010043)
Supplement: Supplementary file 1 [file jof-08-00043-s001.zip › jof-1501170-supplementary.pdf]

Journal of *Fungi*

Supplementary Material

Table S1. Details of the evaluated clinical symptoms. Values attributed to non-metrical parameters such as coughing, breath type, dyspnoea, mucosa aspect, nasal and ocular discharge.

No.	Posture	Breath type	Dyspnoea	Mucosa (conjunctiva)	Ocular discharge	Nasal discharge
1.	normal	costal-abdominal	no dyspnoea	physiological	no ocular discharge	no ocular discharge
2.	altered	abdominal	mildly inspiratory	mildly hyperaemic	mildly serous	mildly serous
3.		costal	moderately inspiratory	moderately hyperaemic	moderately serous	moderately serous
4.			severely inspiratory	severely hyperaemic	severely serous	severely serous
5.			mildly expiratory	mildly anaemic	mildly mucous	mildly mucous
6.			moderately expiratory	moderately anaemic	moderately mucous	moderately mucous
7.			severely expiratory	severely anaemic	severely mucous	severely mucous
8.				mildly cyanotic	mildly purulent	mildly purulent
9.				moderately cyanotic	moderately purulent	moderately purulent
10.				severely cyanotic	severely purulent	severely purulent
11.				mildly icteric	mildly haemorrhagic	mildly haemorrhagic
12.				moderately icteric	moderately haemorrhagic	moderately haemorrhagic
13.				severely icteric	severely haemorrhagic	severely haemorrhagic
14.				oxygen supply mildly reduced		
15.				oxygen supply moderately reduced		
16.				oxygen supply severely reduced		

Table S2. Details of the overall presence of the investigated pulmonary pathogens separated by farms and over time.

	Farm 1					Farm 2					Farm 3					Farm 4					Farm 5				
	1-WO	3-WO	2-MO	3-MO	4-MO	1-WO	3-WO	2-MO	3-MO	4-MO	1-WO	3-WO	2-MO	3-MO	4-MO	1-WO	3-WO	2-MO	3-MO	4-MO	1-WO	3-WO	2-MO	3-MO	4-MO
No infection	0	2	0	0	0	2	0	0	0	0	2	0	0	0	0	1	0	0	0	0	0	0	0	0	0
S.s.	1	0	0	0	0	3	0	0	0	0	0	0	0	0	0	3	0	0	0	0	3	4	0	0	0
G.p. + *S.s.*	6	6	2	0	0	1	3	0	0	0	6	5	0	0	0	4	2	0	0	0	1	4	0	0	0
G.p.	0	1	1	0	0	0	0	0	0	0	0	0	0	0	0	0	0	0	0	0	0	0	0	0	0
B. spp.	1	0	0	0	0	0	0	0	0	0	0	0	0	0	0	0	0	0	0	0	0	0	0	0	0

P. suis + *S.s.*	1	1	0	0	0	0	0	0	0	0	0	0	0	0	0	0	0	0	0	0	3	0	0	0	0
P. suis + *G.p.* + *S.s.*	0	0	2	1	0	0	3	0	0	0	2	0	0	0	0	0	1	1	0	0	0	1	0	0	0
P. suis + *P.m* + *A.p.* + *G.p.* + *S.s.*	0	0	1	0	0	0	0	0	0	0	0	0	0	0	0	0	0	0	0	0	0	0	0	0	0
P. suis + *P.m.* + *G.p.* + *S.s.*	0	0	2	0	0	0	0	0	0	0	0	0	0	0	0	0	0	0	0	0	0	0	1	0	1
P. suis + *G.p.* + *B. spp.* + *M.* spp. + SIV	0	0	0	1	0	0	0	0	0	0	0	0	0	0	0	0	0	0	0	0	0	0	0	0	0
P. suis + *P.m* + *G.p.*+ *S.s.* + *B. spp.* + *M.* spp.	0	0	0	2	1	0	0	0	1	0	0	0	1	0	0	0	0	0	1	1	0	0	0	3	0
P. suis + *G.p.* + *S.s.* + *B. spp.* + *M.* spp.	0	0	0	4	0	0	0	0	0	0	0	0	4	0	0	0	0	0	5	0	0	0	0	1	2
P. suis + *G.p.* + *S.s.* + *M.* spp.	0	0	0	1	0	0	0	3	1	1	0	1	2	0	0	0	2	4	0	1	0	0	4	1	0
P. suis + *P.m.* + *A.p.* + *G.p.* + *S.s.* + *M.* spp.	0	0	0	1	0	0	0	0	0	1	0	0	0	4	1	0	0	0	0	0	0	0	0	0	0
P. suis + *G.p.*+ *S.s.* + *M.* spp. + PCV2	0	0	0	0	1	0	0	0	0	0	0	0	0	0	0	0	0	0	0	0	0	0	0	0	0
P. suis + *G.p.* + *S.s.* + *B. spp.* + *M.* spp. + PCV2	0	0	0	0	1	0	0	0	0	0	0	0	0	0	0	0	0	0	0	0	0	0	0	0	0
P. suis + *P.m* + *G.p.* + *S.s.* + *M.* spp.	0	0	0	0	1	0	0	0	3	0	0	0	0	0	1	0	0	2	0	0	0	0	3	3	0
P. suis + *P.m* + *G.p.* + *S.s.* + *B. spp.* + *M.* spp. + PCV2	0	0	0	0	1	0	0	0	0	0	0	0	0	0	0	0	0	0	0	0	0	0	0	0	0
P. suis + *G.p.* + *M.* spp. + PCV2	0	0	0	0	1	0	0	0	0	0	0	0	0	0	0	0	0	0	0	0	0	0	0	0	0
P. suis + *S.s.* + *M.* spp.	0	0	0	0	0	0	0	0	0	1	0	0	0	0	0	0	0	0	0	0	0	0	0	0	0
P. suis + *G.p.*+ *B. spp.* + *M.*spp.	0	0	0	0	0	0	1	1	0	0	0	0	1	0	1	0	0	0	0	0	0	0	0	0	0
P. suis + *G.p.* + *S.s.* + *B. spp.*	0	0	0	0	0	0	1	0	0	0	0	0	0	0	0	0	0	0	0	0	0	0	1	0	0
P. suis + *G.p.* + *S.s.* + *M.* spp. + SIV	0	0	0	0	0	0	1	0	0	0	0	0	0	0	0	0	0	0	0	0	0	0	0	0	0
P. suis + *A.p.* + *G.p.* + *S.s.* + *B. spp.* + *M.* spp.	0	0	0	0	0	0	0	0	0	0	0	0	0	1	0	0	0	0	0	0	0	0	0	0	0
P. suis + A.p. + *G.p.* + *S.s.* + *M.* spp.	0	0	0	0	0	0	0	0	0	0	0	0	0	4	0	0	0	0	0	0	0	0	0	0	0
P. suis + *P.m* + *A.p.* + *G.p.* + *S.s.* + *B. spp.* + *M.* spp.	0	0	0	0	0	0	0	0	0	0	0	0	0	1	0	0	0	0	0	0	0	0	0	1	0
P. suis + *S.s.* + *B. spp.*	0	0	0	0	0	0	0	0	0	0	0	0	0	0	0	0	0	0	0	0	1	0	0	0	0
P. suis + *G.p.* + *S.s.* + *B. spp.* + *M.* spp. + SIV	0	0	0	0	0	0	0	4	0	0	0	0	0	0	0	0	0	0	0	0	0	0	0	0	0
P. suis + P.m. + *G.p.* + *S.s.* + *B. spp.* + M. spp. + SIV	0	0	0	0	0	0	0	2	0	0	0	0	0	0	0	0	0	0	0	0	0	0	0	0	0
P. suis + *G.p.* + *M.* spp.	0	0	0	0	0	0	0	0	0	0	0	0	0	0	1	0	0	1	0	1	0	0	0	0	0
P. suis + *G. p.*	0	0	0	0	0	0	0	0	0	0	0	0	0	0	0	0	0	1	0	0	0	0	0	0	0
P. suis	0	0	0	0	0	0	0	0	0	0	0	0	0	0	0	0	0	0	0	0	1	1	0	0	0

P. suis + *P.m.* + *G.p.* + *S.s.* + *M.* spp. + SIV	0	0	0	0	0	0	0	0	0	0	0	0	0	0	0	0	0	0	0	0	0	0	1	0	0
P. suis + *P.m* + *G.p.* + *S.s.* + *B. spp.*	0	0	0	0	0	0	0	0	0	0	0	0	0	0	0	0	0	0	1	0	0	0	0	0	0
P. suis + *G.p.* + *B. spp.*	0	0	0	0	0	0	0	0	0	0	0	0	0	0	0	0	0	0	2	0	0	0	0	0	0
G.p. + *M.* spp.	0	0	0	0	1	0	0	0	0	0	0	0	0	0	2	0	0	0	0	0	0	0	0	0	0
G.p. + *S.s.* + *B. spp.*	0	0	0	0	0	1	0	0	0	0	0	0	0	0	0	0	0	0	0	0	0	0	0	0	0
G.p. + *S.s.* + *M.* spp.	1	0	0	0	0	0	1	0	3	2	0	0	1	0	0	2	4	0	0	1	0	0	0	0	0
G.p. + *S.s.* + *B. spp.* + *M.* spp.	0	0	0	0	1	0	0	0	0	0	0	0	0	0	0	0	0	0	1	1	0	0	0	0	2
G.p. + *S.s.* +*M.* spp. + PCV2	0	0	0	0	2	0	0	0	0	1	0	0	0	0	0	0	0	0	0	0	0	0	0	0	0
G.p. + *S.s.* + PCV2	0	0	0	0	0	0	0	0	0	0	0	1	0	0	0	0	0	0	0	0	0	0	0	0	0
P.m + *G.p.* + *S.s.*	0	0	2	0	0	2	0	0	0	0	0	3	0	0	0	0	0	0	0	0	0	0	0	0	1
P.m + *G.p.* + *S.s.* + *B. spp.* + *M.* spp.	0	0	0	0	0	0	0	0	0	0	0	0	1	0	1	0	0	0	0	0	0	0	0	0	0
P.m + *G.p.* + *S.s.* + *M.* spp.	0	0	0	0	0	0	0	0	1	3	0	0	0	0	0	0	1	0	0	1	0	0	0	1	0
P.m + *M.* spp.	0	0	0	0	0	0	0	0	0	0	0	0	0	0	0	0	0	0	0	1	0	0	0	0	0
P.m + *G.p.* + *M.* spp.	0	0	0	0	0	1	0	0	0	0	0	0	0	0	0	0	0	0	0	0	0	0	0	0	0
P.m + *A.p.* + *G.p.* + *S.s.* + *M.* spp.	0	0	0	0	0	0	0	0	0	0	0	0	0	0	0	0	0	0	0	0	0	0	0	0	1
P.m + *A.p.* + *G.p.* + *S.s.* + *B. spp.* + *M.* spp.	0	0	0	0	0	0	0	0	0	0	0	0	0	0	0	0	0	0	0	0	0	0	0	0	1
P.m + *G.p.*	0	0	0	0	0	0	0	0	0	0	0	0	0	0	0	0	0	0	0	0	0	0	0	0	1
P.m + *S.s.*	0	0	0	0	0	0	0	0	0	0	0	0	0	0	0	0	0	0	0	0	1	0	0	0	0
A.p. + *G.p.* + *S.s.* + *B. spp.* + *M.* spp.	0	0	0	0	0	0	0	0	0	0	0	0	0	0	1	0	0	0	0	0	0	0	0	0	0
A.p. + *G.p.* + *S.s.* + *M.* spp.	0	0	0	0	0	0	0	0	0	0	0	0	0	0	1	0	0	0	0	3	0	0	0	0	1
A.p. + *G.p.* + *M.* spp.	0	0	0	0	0	0	0	0	0	0	0	0	0	0	1	0	0	0	0	0	0	0	0	0	0
S.s. + *M.* spp.	0	0	0	0	0	0	0	0	0	1	0	0	0	0	0	0	0	0	0	0	0	0	0	0	0
**Sum**	**10**	**10**	**10**	**10**	**10**	**10**	**10**	**10**	**9**	**10**	**10**	**10**	**10**	**10**	**10**	**10**	**10**	**9**	**10**	**10**	**10**	**10**	**10**	**10**	**10**

P. suis: *Pneumocystis suis*; SIV: Swine Influenza Virus; *A.p.*: *Actinobacillus pleuropneumoniae*; *B.* spp.: *Bordetella* spp. (*B. bronchiseptica, B. pertussis* and *B. parapertussis*); *M.* spp.: *Mycoplasma* spp. (*M. hyopneumoniae, M. hyorhinis* and *M. flocculare*); *P.m.*: *Pasteurella multocida; G.p.*: *Glaesserella parasuis; S.s.*: *Streptococcus suis.*

Table S3. Details of possible associations between *P. suis* and the various pathogen combinations on farm level.

Farm	Pathogen combination	*ρ*	*p*
Farm 1	*P. suis* + *S.s.*	-0.889	0.044
Farm 1	*P. suis* + *B.* spp. + *G.p.* + *M.* spp. + *P. m.* + *S.s.*	0.918	0.028
Farm 4	*P. suis* + *G.p.* + *M.* spp. + *S.s.*	0.889	0.044
Farm 5	*P. suis* + *G.p.* + *M.* spp. + *S.s.*	0.968	0.007
Farm 5	*P. suis* + *G.p.* + *M. spp.* + *P.m.* + *S.s.*	0.968	0.007

P. suis: *Pneumocystis suis*; *B.* spp.: *Bordetella* spp. (*B. bronchiseptica, B. pertussis* and *B. parapertussis*); *M.* spp.: *Mycoplasma* spp. (*M. hyopneumoniae, M. hyorhinis* and *M. flocculare*); *P.m.*: *Pasteurella multocida; G.p.*: *Glaesserella parasuis; S.s.*: *Streptococcus suis.*
ρ = Spearman's coefficient of correlation, p = p-value or probability value.

Table S4. Details of significant correlations between pulmonary pathogens and clinical symptoms in samples sorted by time.

Time point	Symptom	Pathogen combination	ρ	p
1-WO	Cough	*P. suis* + *S.s.*	0.918	0.028
3-WO	Cough	*P. suis* + *G.p.* + *S.s.*	0.913	0.03
3-WO	Nasal discharge	*P.suis*+*G.p.*+ *M.* spp. + *S.s.*	0.894	0.041
2-MO	Weight	*P. suis* + *G.p.* + *M.* spp. + *P.m.* + *S.s.*	0.894	0.041
3-MO	Nasal discharge	*P. suis positive samples*	0.973	0.005
3-MO	Dyspnoea	*P. suis* + *B.* spp. + *G.p.* + *M.* spp. + *P.m* + *S.s.*	0.918	0.026
4-MO	Cough	*G.p.* + *M.* spp. + *P.m.* + *S.s.*	0.884	0.047
4-MO	Cough	*G.p.*+ *M.* spp. + *S.s.*	0.884	0.047
4-MO	Cough	*P. suis* + *G.p.* + *M.* spp.+ *S.s.*	0.913	0.03

P. suis: *Pneumocystis suis*; *B.* spp.: *Bordetella* spp. (*B. bronchiseptica, B. pertussis* and *B. parapertussis*); *M.* spp.: *Mycoplasma* spp. (*M. hyopneumoniae, M. hyorhinis* and *M. flocculare*); *P.m.*: *Pasteurella multocida*; *G.p.*: *Glaesserella parasuis*; *S.s.*: *Streptococcus suis*.
ρ = Spearman's coefficient of correlation, p = p-value or probability value.

Table S5. Details of significant correlations between pulmonary pathogens and clinical symptoms in samples sorted by farms.

Farm	Symptom	Pathogen combination	ρ	p
Farm 2	Breath frequency	*P. suis* + *G.p.* + *M.* spp. + *S.s.*	0.949	0.014
Farm 5	Nasal discharge	*P. suis* + *G.p.* + *P.m.* + *S.s.*	0.884	0.047
Farm 5	Dyspnoea	*P. suis*	0.889	0.044
Farm 5	Weight	*S.s.*	0.894	0.041
Farm 5	Weight	*G.p.* + *S.s.*	0.894	0.041

P. suis: *Pneumocystis suis*; *M.* spp.: *Mycoplasma* spp. (*M. hyopneumoniae, M. hyorhinis* and *M. flocculare*); *P.m.*: *Pasteurella multocida*; *G.p.*: *Glaesserella parasuis*; *S.s.*: *Streptococcus suis*.
ρ = Spearman's coefficient of correlation, p = p-value or probability value.
